# New Partners, New Tools, New Possibilities: Views From the Fields of Education and Public Health

**Published:** 2007-06-15

**Authors:** Michael Schmoyer

**Affiliations:** Office of the Director, Division of Adolescent and School Health, National Center for Chronic Disease Prevention and Health Promotion, Centers for Disease Control and Prevention

School-based programs should be a key component of a comprehensive community health promotion agenda. Youth who are ill, are physically inactive, use tobacco, or use other drugs are unlikely to succeed in school. Furthermore, the burden that chronic diseases place on our nation's medical and economic systems will likely worsen unless we in public health are able to prevent the risk among our young people (1). Our nation's 121,000 schools can play a critically important role in improving the health of children and adolescents. School-based health promotion programs also can have a positive impact on the academic performance, quality of life, and economic productivity of students (2).

The Division of Adult and Community Health (DACH) of the Centers for Disease Control and Prevention (CDC) recently brought together 25 individuals as the National Expert Panel on Community Health Promotion to get their recommendations on enhancing community health promotion (3). At least two recommendations are relevant for school health programs: 1) build capacity for community health promotion, and 2) promote training and capacity building that give the public health workforce the knowledge, skills, and tools to implement effective community health promotion approaches.

CDC currently funds state education and health agencies, large urban school districts, and national nongovernmental organizations to build the capacity of schools to implement effective health promotion policies and programs. These programs address priority health risks among youth, including tobacco use, poor nutrition, physical inactivity, obesity, and human immunodeficiency virus infection and other sexually transmitted diseases.

Through CDC's collaborations (both funded and nonfunded) with schools and their partners, many capacity-building projects have been successfully implemented. CDC has a system of professional development and training that works with national nongovernmental partners to provide technical assistance and resources to state education and health agencies. These agencies assist large urban districts in establishing effective coordinated school health programs. In turn, school districts work with their schools to strengthen their capacity for improving the delivery, effectiveness, and sustainability of disease prevention and health promotion programs for youth. Mechanisms for capacity building have included training, peer-to-peer learning, consulting on technology skills, and building evaluation skills.

CDC also has developed a number of evidence-based tools that the public health workforce can use to implement effective community health promotion approaches in schools. Each tool builds on the recommendations found in CDC guidelines for school health programs, which are developed based on a rigorous review of evaluations of school-based health promotion interventions and input from a broad cross section of researchers and practitioners. CDC's school health promotion tools include the *School Health Index: A Self-Assessment and Planning Guide*; *Fit, Healthy, and Ready to Learn: A School Health Policy Guide*; and *Making It Happen: School Nutrition Success Stories* (4). These user-friendly tools can be used effectively by public health professionals, educators, and parent and community volunteers. They have been used by thousands of schools across the nation.

The key to the success of CDC's Division of Adolescent and School Health and school-based health in general is the partnership between health and educational agencies and CDC's recognition and understanding of the school's role in the larger social structure of communities. Public health employees, educators, and community members who work together can more effectively improve family and community structures and thereby reduce risks associated with chronic diseases. Through this shared learning and expertise, local schools can implement effective policies and programs. CDC is playing a critical role by supporting capacity building and promoting training for education partners who, in turn, affect the lives of millions of children each day.
